# Exploring the link between Multimorbidity and direct healthcare costs in Ireland: A cross-sectional study

**DOI:** 10.1177/26335565231219421

**Published:** 2023-12-10

**Authors:** Sharon Walsh, Paddy Gillespie

**Affiliations:** 1 School of Business and Economics, 8799 University of Galway, Galway, Ireland

**Keywords:** Multimorbidity, direct healthcare costs, cost analysis

## Abstract

**Background:**

Multimorbidity has emerged as a major challenge facing health services globally, which will place a substantial burden on health systems going forward. This paper seeks to estimate the association between multimorbidity and direct healthcare costs among older people in Ireland from a healthcare system perspective.

**Methods:**

Cross-sectional analysis of data on 8,447 community-dwelling adults aged 50 and over collected between 2009 and 2011 as part of the Irish Longitudinal Study on Ageing. Multivariable generalised linear model regression, employing a log-link and Poisson family distribution, is used to assess the association between self-reported multimorbidity status and direct healthcare costs.

**Results:**

For the full sample, 21.20% reported having no chronic conditions, 27.39% had one chronic condition, and 51.40% had multimorbidity. After controlling for a range of socio-demographic and health status variables, we found that relative to those reporting no chronic conditions, one chronic condition was associated with additional average annual costs of €513 (95% CIs: 245, 781), increasing to €1277 (95% CIs: 942, 1612) for those with 6 or more chronic conditions. Relative to those reporting 2 chronic conditions, 4 chronic conditions were associated with additional costs of €411 (95% CIs: 106, 716), 5 chronic conditions with €591 (95% CIs: 214, 969), and 6 or more chronic conditions with additional average costs of €1006 (95% CIs: 641, 1371).

**Conclusion:**

This study finds positive and significant associations between the number of chronic conditions and direct healthcare costs and further highlights the potential economic benefits from preventing the onset and progression of multimorbidity

## Introduction

Multimorbidity, commonly defined as the co-existence of two or more chronic conditions in the same individual, has emerged as a significant public health challenge globally.^1-4^ Recent estimates from multi-country systematic reviews suggest that approximately one-third of adults have multimorbidity, with prevalence rising to over 50% in those aged 60 and over.^5, 6^ Thus, while it is not limited to older people, multimorbidity is progressively more common with age. Given this, the prevalence of multimorbidity is expected to rise in the coming years, as many countries experience population ageing.^7-9^ This expected increase in the number of people with multimorbidity will place significant pressures on health systems, as multimorbidity is associated with a decline in health status, functional capacity and quality of life.^10-12^

Indeed, multimorbidity is associated with increased healthcare utilisation and costs, and this burden increases as the number of co-occurring chronic conditions increases.^13,14^ In a systematic review of health care utilisation and costs among older people with multimorbidity, Lehnert, Heider^15^ found that both utilisation and costs significantly increased with each additional chronic condition in almost all studies. In fact, several of the included studies observed a near exponential relationship, in which expenditures about doubled with each additional chronic condition.^15^ More recently, Soley-Bori, Ashworth^16^ conducted a systematic review of the impact of multimorbidity on healthcare costs and utilisation in the UK. They found that multimorbidity generally translates to increased utilisation and costs across a wide range of resource categories, including primary care, dental care, emergency department care, transition care, and hospital care. Indeed, the most sizeable impact of multimorbidity is on unplanned, potentially preventable, hospitalisations, with up to 14.38 times increased odds for those with four or more conditions.^16^

In Ireland, the country of relevance to this study, Glynn, Valderas,^17^ adopting a healthcare system perspective, estimated the additional direct healthcare costs associated with multimorbidity in 2011. In a sample of 3309 patients in the community, the prevalence of multimorbidity was 66.2% in those over 50 years of age. Primary and hospital care utilization and costs were found to be significantly higher among patients with multimorbidity status, which was analysed using a count variable ranging from zero conditions to four or more conditions. After adjusting for age, gender and free medical care eligibility, the addition of each chronic condition of up to four or more conditions was associated with an increase in primary care consultations, hospital outpatient visits, hospital admissions and total health care costs. More recently, Larkin, Walsh ^18^, adopting a patient perspective and using data for a sample of 5899 individuals from wave 4 of the Irish Longitudinal Study on Ageing (TILDA), estimated the additional out-of-pocket expenses associated with multimorbidity. They found that having multimorbidity, which was analysed using a count variable ranging from zero conditions up to 3 or more conditions, increased an individual’s likelihood of having out-of-pocket healthcare expenditure by over 20%. Even after controlling for several sociodemographic factors, they showed that having multimorbidity was associated with a large absolute increase in out-of-pocket healthcare expenditure. Interestingly, individuals with multimorbidity were also found to have substantially lower equivalised household income than individuals with no conditions.^18^ Our work builds upon this earlier research by exploring from the healthcare system perspective, the relationship between multimorbidity, which is analysed using a count variable ranging from zero conditions up to 6 or more conditions, and direct formal healthcare costs. We examine this, first, relative to those with no chronic conditions, and second, relative to those with two chronic conditions, that is, within the multimorbidity cohort.

Thus, multimorbidity represents an important economic and societal challenge, which health policy makers, and health and social care providers will need to address directly. Given this, evidence on the impact of multimorbidity on healthcare utilisation and costs will be useful for informing future health policy and care delivery. Notably, the needs of multimorbidity patients are different from those with a single chronic disease and may differ depending on the mix of chronic conditions experienced. Indeed, guidelines suggest that they should be offered individualised care plans, delivered by a range of health and social care professionals.^5,14,19^ Moreover, while primary and community care has been highlighted as an ideal setting for such care plans, recent systematic reviews of interventions for multimorbidity found limited evidence of their health benefit or cost effectiveness.^20,21^ Reviewers concluded that interventions may be more effective if focused on risk factors common across co-morbid conditions or generic outcomes such as daily functioning.^20,21^ In Ireland, this evolving policy and practice landscape has translated into new initiatives such as the establishment of a structured chronic disease management programme, which seeks to improve primary care management of chronic diseases.^22^ While this has been an important addition to chronic disease and multimorbidity policy and clinical practice in Ireland, the future sustainability of such programmes of care in an already resource constrained health system will depend upon their resultant impacts on health outcomes, costs and cost effectiveness.

To this end, a variety of data on effectiveness, health outcomes, costs and preferences will be required to build the evidence base for future health technologies, as well as health and social care programmes and interventions, which target multimorbidity. Within this context, this paper seeks to add to the existing evidence base by further exploring the relationship between multimorbidity, measured by the number of chronic conditions experienced, and direct healthcare costs for community dwelling older adults in Ireland from a healthcare system perspective.

## Methods

### Sample

Data from the first wave of TILDA, which was collected between 2009 and 2011, were used for the cross-sectional analysis. While later waves of TILDA are available for analysis, wave 1 was employed as it has the largest sample size available for analysis, which allows for a more comprehensive categorisation of multimorbidity status than existing Irish studies (that is, up to 6 or more conditions). It also provides the necessary count data for the healthcare utilisation variables, which are used to generate the total healthcare cost variable. TILDA is a nationally representative study of community-dwelling adults aged 50 years and over in the Republic of Ireland. Detail on the sampling and data collection methods used in TILDA can be found elsewhere.^23^ In brief, the first wave of TILDA was a two-stage clustered survey, including a random sample of 640 clusters of 500 to 1,180 residential addresses which was selected nationally using a sampling frame stratified according to socioeconomic group and geography. Clusters were randomly selected, with a probability of selection proportionate to the proportion of the population in each cluster aged 50 and older.^23^ Forty addresses in each cluster were randomly selected, and all persons aged 50 and older were asked to participate in the study. During fieldwork, 8,504 interviews were conducted with individuals in 6,279 households, with a response rate of 62%. Interviews were conducted in respondents’ homes using a computer-aided personal interview. A self-completion questionnaire with more-sensitive questions was left for the respondent to complete and return. For the purpose of our analysis, we excluded participants for whom we had no healthcare utilisation or control variable response data, which results in a final estimation sample of 8,447 TILDA participants from the full sample of 8,504 participants. Descriptive statistics for the sample are presented in Table 1.

**Table 1.** Variable Descriptions and Descriptive Statistics for Study Sample and Subsample.

Variable Name	Variable Description	Full Estimation Sample	Two or More Chronic Conditions
**N (%)**		8,447 (100)	4342 (51.40)
**Chronic Condition Status**		**N (%)**	**N (%)**
	No chronic conditions	1,791 (21.20)	***
	1 chronic condition	2,314 (27.39)	***
	2 chronic conditions	1,871 (22.15)	1,871 (43.09)
	3 chronic conditions	1,171 (13.86)	1,171 (26.97)
	4 chronic conditions	688 (8.14)	688 (15.85)
	5 chronic conditions	329 (3.89)	329 (7.58)
	6 or more chronic conditions	283 (3.35)	283 (6.52)
**Other Characteristics**		**N (%)**	**N (%)**
*Age Category*	<55 years	1,946 (23.04)	626 (14.42)
	55 – 65 years	3,022 (35.78)	1,457 (33.56)
	65 – 75 years	2,146 (25.41)	1,353 (31.16)
	75 – 85 years	1,104 (13.07)	766 (17.64)
	> 85 years	229 (2.71)	140 (3.22)
*Gender*	Male	4,691 (55.53)	1,832 (42.19)
	Female	3,756 (44.47)	2,510 (57.81)
*Marital Status*	Married or Cohabiting	5,927 (70.17)	2,863 (65.94)
	Other	2,520 (29.8)	1,479 (34.06)
Education Level	Primary Education	2,495 (29.54)	1,467 (33.79)
	Secondary Education: Intermediate	1,959 (23.19)	972 (22.39)
	Secondary Education: Leaving Certificate	1,453 (17.20)	673 (15.50)
	Third Level: Diploma/Certificate	1,331 (15.76)	667 (15.36)
	Third Level: Primary Degree	728 (8.62)	348 (8.01)
	Third Level: Postgraduate/Higher Degree	481 (5.69)	215 (4.95)
*Employment*	Retired	3,025 (35.81)	1,897 (43.69)
	Employed	3,129 (37.04)	1,160 (26.72)
	Other	2,293 (27.15)	1,285 (29.59)
*Medical Card*	No Medical Card	4,355 (51.56)	1,795 (41.34)
	Full Medical Card	3,947 (46.73)	2,471 (56.91)
	GP Visit Medical Card	145 (1.72)	76 (1.75)
*Private Health Insurance*	No Private Health Insurance	3,561 (42.16)	1,901 (43.78)
	Private Health Insurance	4,886 (57.84)	2,441 (56.22)
*Region*	Dublin City or County	2,005 (23.74)	1,120 (25.79)
	Other Town or City	2,377 (28.14)	1,239 (28.54)
	Rural Area	4,065 (48.12)	1,983 (45.67)
*Nationality*	Irish	7,637 (90.41)	3,925 (90.40)
	Non-Irish	810 (9.59)	417 (9.60)
*Living Arrangement*	Living Alone	1,799 (21.30)	1,079 (24.85)
	Living with Spouse	3,280 (38.83)	1,807 (41.62)
	Living with Others	3,368 (39.87)	1,456 (33.53)
*Self-Rated Health*	Excellent	1,357 (16.06)	322 (7.42)
	Very Good	2,437 (28.85)	950 (21.88)
	Good	2,740 (32.44)	1,566 (36.07)
	Fair	1,500 (17.76)	1,129 (26.00)
	Poor	413 (4.89)	375 (8.64)

### Dependent variable

For the purpose of the cost analysis, which adopts a healthcare system perspective, a healthcare cost variable was generated by valuing self-reported resource use data for general practitioner (GP) visits, hospital inpatient admissions, outpatient clinic visits, and accident and emergency (A&E) department visits. Utilisation of community and social care data was not collected in count form as part of the survey, and data on medications was not collected in a form that allowed for direct costing for analysis. Therefore, for pragmatic reasons, we did not include these resources in the healthcare cost variable, which is a key limitation of the study. Resource use data were captured over a 12-month period, while the cost data were generated by multiplying resource use by the respective unit cost, and summing these into a combined total healthcare cost estimate.^1^ One notable limitation of this approach was that self-reported resource use for each service was truncated in the publically available wave 1 TILDA database. That is, GP visits were truncated in the dataset at a maximum value of 25; inpatient admissions were truncated at 6; outpatient visits were truncated at 10; and A&E visits were truncated at 6. However, as the proportion of respondents located in the highest truncated resource use categories was minimal (i.e. < 1% for GP, inpatient and A&E usage, and 2.9% for outpatient usage), our findings are unlikely to be significantly impacted, although our cost results are likely to be underestimates. Unit cost data were derived from a number of public data sources and were inflated to 2020 prices using the health component of the consumer price index^24^ – please see Appendix 4. In particular, the unit cost for GP consultations was obtained from a published study by Smith, Jiang,^25^ which presents unit cost estimates for a range of primary and community care services in Ireland. The unit cost estimate was presented originally in 2019 € prices and was generated from a national survey of private GP fees. Unit cost estimates for hospital services including inpatient admissions, outpatient clinic consultations and accident and emergency department visits were obtained via a direct request to the Hospital Pricing Office (HPO) in Ireland. The unit cost estimates were presented originally in 2015 € prices and were generated using the HPO’s case-mix and diagnosis related group (DRG) costing methodology.

### Independent variables

The key explanatory variable in this analysis relates to the chronic disease and multimorbidity status of individuals in the sample. TILDA respondents were provided with a list of conditions and were asked if a doctor had ever diagnosed them with any of them (see Appendix 1 for the survey questions). For this study, a chronic disease and multimorbidity status variable was pragmatically generated based on a count of 23 self-reported chronic conditions. In line with other studies, we define multimorbidity as the co-existence of two or more chronic conditions.^13,18^ Please see Appendix 3 for details on the chronic disease profile of the estimation sample. For the purposes of the statistical analysis, a multimorbidity status count variable with the following categories was generated: (i) no chronic conditions; (ii) one chronic condition; (iii) two chronic conditions; (iv) three chronic conditions; (v) four chronic conditions; (vi) five chronic conditions; (vii) 6 or more chronic conditions. Please see Table 1 for the multimorbidity status of the estimation sample.

A number of other variables were used as independent control variables in the model. Sample descriptive statistics for these are presented in Table 1, and details of the survey questions are provided in Appendix 1. As per the Andersen model, we assume that healthcare utilisation is influenced by a range of predisposing factors (e.g. age, gender, education, occupation and ethnicity), enabling factors (e.g. health insurance status), and need factors (e.g. perceived and actual health status), and we attempt to capture these influences in our selection of control variables.^26^ In particular, a range of sociodemographic and education-related variables were included. Age is included as a categorical variable, ranging from under 55 years old to over 85 years old. Gender is included as a binary variable – male or female. Marital status is also included as a binary variable, which is equal to 1 if married or cohabiting, and equal to 0 if single, divorced, widowed or separated (“other”). Education level is included as a categorical variable, ranging from primary education to a third level postgraduate education. Employment is also captured as a categorical variable, equal to 1 if retired, 2 if employed and 3 if unemployed, permanently sick or disabled, looking after the home, or in education (“other”). In addition to these socio-demographic controls, variables capturing the region, nationality, and living arrangements of the respondents were also included. Health insurance status is captured using public medical card status and private health insurance status. Respondents were asked to indicate whether or not they are covered by a medical card, which may be viewed a form of public health insurance in Ireland. Medical cards entitle individuals to free primary, community and public hospital care, as well as lower prescription co-payments. Eligibility for a medical card is primarily based on a means test, with those in the lowest income groups entitled to a card.^27^ If you are not eligible for a medical card on income grounds, you may be eligible for a GP visit card, which allows you to visit a GP for free.^28^ All adults aged over 70 are entitled to GP visit cards regardless of income. Related to this, respondents were asked if they had private health insurance (PHI) cover. PHI is voluntary in Ireland, and the majority of PHI plans are focussed on providing access to private hospitals and/or private rooms in public hospitals.^29^ Finally, a variable capturing the self-rated health of the respondents were included.

### Statistical analysis

A series of descriptive and regression analyses were undertaken. For the descriptive analysis, means, medians, standard deviations and interquartile ranges were used to summarise healthcare resource use and healthcare costs. For the multivariable cost analysis, a generalised linear model (GLM) regression approach was adopted. This is widely used in the analysis of healthcare cost data,^30-32^ as it allows for the skewed nature of cost data.^33^ The choice of distribution was informed by a Modified Park test, and the log link function was informed by a Pearson correlation test, a Pregibon link test and a Modified Hosmer and Lemeshow test. This approach has been shown to be appropriate for the analysis of cost data.^34,35^

The following regression models were estimated. First, a univariate cost regression model was estimated controlling for *multimorbidity status* only for the full estimation sample (n=8,447). Multimorbidity status was included as a seven category variable: (i) no chronic conditions, the reference category; (ii) one chronic condition; (iii) two chronic conditions; (iv) three chronic conditions; (v) four chronic conditions; (vi) five chronic conditions; (vii) 6 or more chronic conditions. Second, a multivariable cost regression model was estimated controlling for *multimorbidity status*, in addition to *age, gender, marital status, educational status, employment status, medical card status, private health insurance status, region, nationality and self-reported health status*. Third, univariate and multivariable regression models were estimated for the multimorbidity cohort sample (n=4,342). The reference category was two chronic conditions, which allows us to examine the impact of additional chronic conditions on those with existing multimorbidity. Finally, we undertake a number of supplemental multivariable analysis for the full estimation sample (n=8,447). We examine the association between multimorbidity status and the respective costs of GP consultations, hospital inpatient admissions, outpatient clinic consultations, and accident and emergency department visits. Finally, we conduct separate analysis for males and females, and for each age category. Statistical significance was explored at the 0.05 level and model fit by the Akaike information criterion (AIC). All analyses was performed using Stata 15.0.^36^

## Results

Table 1 presents descriptive statistics for the number of chronic conditions, as well as a range of socio-demographic and health status variables. For the full estimation sample, 1,791 (21.20%) reported having no chronic conditions, 2,314 (27.39%) had one chronic condition, and 4,342 (51.40%) had two or more chronic conditions. Descriptive statistics for healthcare resource use and healthcare costs are presented in Table 2. For the full estimation sample, the mean (standard deviation (SD)) number of GP visits was 3.80 (SD: 4.10), hospital inpatient admissions was 0.19 (SD: 0.63), outpatient visits was 1.19 (SD: 2.18), and A&E visits was 0.22 (SD: 0.67). With regard to total healthcare costs, the mean healthcare cost was €1283 (SD: 3118) for the full estimation sample. For those with no chronic conditions (n=1791), the mean healthcare cost was €548 (SD: 2017). For those with one chronic condition (n=2,314), the mean healthcare cost was €975 (SD: 2855). For those with two or more chronic conditions (n=4342), the mean healthcare cost was €1749 (SD: 3521).

**Table 2.** Descriptive Statistics for Health Service Use and Healthcare Cost by Chronic Disease Status.

Variable		N	Mean	Median	Standard Deviation	IQR (Q1-Q3)
Resource Use Item						
GP Visits	Full Sample	8,447	3.80	3	4.10	1-5
	Two or More Chronic Conditions	4,342	5.02	4	4.57	2-6
INPT Admissions	Full Sample	8,447	0.19	0	0.63	0-1
	Two or More Chronic Conditions	4,342	0.26	0	0.71	0-0
OPD Visits	Full Sample	8,447	1.19	0	2.18	0-1
	Two or More Chronic Conditions	4,342	1.68	1	2.52	0-2
AE Visits	Full Sample	8,447	0.22	0	0.67	0-0
	Two or More Chronic Conditions	4,342	0.29	0	0.77	0-0
**Health Care Cost**						
	Full Sample	8,447	€1283	€238	€3118	€102-€663
	Two or More Chronic Conditions	4,342	€1749	€417	€3521	€187-€1071
	No chronic conditions	1,791	€548	€51	€2017	€0-€238
	1 chronic condition	2,314	€975	€187	€2855	€51-€442
	2 chronic conditions	1,871	€1285	€272	€2935	€102-€706
	3 chronic conditions	1,171	€1557	€391	€3123	€204-€987
	4 chronic conditions	688	€2087	€578	€3860	€255-€1463
	5 chronic conditions	329	€2643	€748	€4374	€391-€4810
	6 or more chronic conditions	283	€3762	€1122	€5278	€544-€5711

Note: GP refers to General Practitioner. INPT refers to in-patient hospital admissions. OPD refers to hospital outpatient consultant visits. AE refers to Accident and Emergency department visits.

The results for the GLM analyses of costs are presented in Tables 3 and 4, and are summarised below. In all cases, a GLM regression assuming a Poisson distribution and a log link function was the preferred approach. In the multivariable analysis for the full estimation sample (Table 3), chronic disease status, gender, education status, medical card status, private health insurance status, and self-reported health status were significantly associated with healthcare costs. Relative to those reporting no chronic conditions, a single chronic condition was associated with additional average costs of €513 (95% CIs: 245, 781), 2 chronic conditions €610 (95% CIs: 346, 873), 3 chronic conditions €689 (95% CIs: 401, 977), 4 chronic conditions €861 (95% CIs: 559, 1163), 5 chronic conditions €997 (95% CIs: 651, 1343), and 6 or more chronic conditions €1277 (95% CIs: 942, 1612)

**Table 3.** Marginal effect coefficients from generalized linear regression models of healthcare costs (€), estimated using a Poisson error distribution and a log link function, based on the full estimation sample.

Variable Name	Variable Description	Univariate Model € Coeffi (SE) (P-Value) (95% CIs)	Multivariable Model € Coeffi (SE) (P-Value) (95% CIs)
Chronic Condition Status	No chronic conditions 1 chronic condition 2 chronic conditions 3 chronic conditions 4 chronic conditions 5 chronic conditions 6 or more chronic conditions	*Ref* 734 (138) (0.000) (469, 1010) 1093 (133) (0.000) (833, 1353) 1339 (138) (0.000) (1070, 1609) 1715 (150) (0.000) (1422, 2009) 2019 (169) (0.000) (1686, 2351) 2471 (167) (0.000) (2144, 2798)	*Ref* 513 (137) (0.000) (245, 781) 610 (135) (0.000) (346, 873) 689 (147) (0.000) (401, 977) 861 (154) (0.000) (559, 1163) 997 (177) (0.000) (651, 1343) 1277 (171) (0.000) (942, 1612)
Age Category	<55 years 55 – 65 years 65 – 75 years 75 – 85 years > 85 years		*Ref* 125 (98) (0.205) (-68, 317) 226 (129) (0.079) (-26, 478) 229 (145) (0.113) (-54, 512) 83 (234) (0.722) (-376, 542)
Gender	Male		151 (69) (0.028) (16, 285)
	Female		*Ref*
Marital Status	Married or Cohabiting		-17 (133) (0.898) (-277, 243)
	Other		*Ref*
Education Level	Primary Education Secondary Education: Intermediate Secondary Education: Leaving Certificate Third Level: Diploma/Certificate Third Level: Primary Degree Third Level: Postgraduate/Higher Degree		*Ref* 235 (95) (0.014) (49, 422) 291 (106) (0.006) (84, 498) 443 (112) (0.000) (224, 664) 434 (131) (0.001) (178, 690) 391 (169) (0.021) (60, 721)
Employment	Retired		*Ref*
	Employed		-140 (101) (0.164) (-338, 57)
	Other		99 (92) (0.283) (-82, 280)
Medical Card	No Medical Card		*Ref*
	Full Medical Card		340 (88) (0.000) (167, 513)
	GP Visit Medical Card		192 (206) (0.351) ( -211, 595)
Private Health Insurance	No Private Health Insurance		*Ref*
	Private Health Insurance		184 (77) (0.017) (32, 335)
Region	Dublin City or County		*Ref*
	Another Town or City Rural Area		136 (88) (0.123) (-37, 308) 112 (82) (0.171) (-48, 271)
Nationality	Irish		13 (112) (0.905) (-205, 232)
	Non-Irish		*Ref*
Living Arrangement	Living Alone		*Ref*
	Living with Spouse		-50 (158) (0.750) (-360, 260)
	Living with Others		-9 (130) (0.944) (-263, 245)
Self-Rated Health	Excellent		*Ref*
	Very Good		271 (143) (0.059) (-10, 552)
	Good		753 (147) (0.000) (465, 1042)
	Fair		1455 (156) (0.000) (1149, 1760)
	Poor		1971 (182) (0.000) (1615, 2327)
N GLM Family/Link AIC Modified Park Test Chi_Squared p-value Pearson Correlation Test Pregibon Link Test Modified Hosmer and Lemeshow		8,447 Poisson/Log 16.06635 0.925474 0.6377 1.0000 1.0000 1.0000	8,447 Poisson/Log 2854.857 1.098922 0.4698 0.8913 0.6422 0.3881

***p<0.01, ** p<0.05, *p<0.10.

**Table 4.** Marginal effect coefficients from generalized linear regression models of healthcare costs (€), estimated using a Poisson error distribution and a log link function, based on the multimorbidity (i.e. two or more chronic conditions) estimation sample.

Variable Name	Variable Description	Univariate Model € Coeffi (SE) (P-Value) (95% CIs)	Multivariable Model € Coeffi (SE) (P-Value) (95% CIs)
Chronic Condition Status	2 Chronic Conditions 3 Chronic Conditions 4 Chronic Conditions 5 Chronic Conditions 6 or More Chronic Conditions	*Ref* 336** (138) (0.015) (65, 607) 849*** (157) (0.000) (542,1156) 1263*** (189) (0.000) (892, 1633) 1880*** (183) (0.000) (1521, 2239)	*Ref* 144 (141) (0.306) (-132, 419) 411** (155) (0.008) (106, 716) 591*** (193) (0.002) (214, 969) 1006*** (186) (0.000) (641, 1371)
Age Category	<55 years 55 – 65 years 65 – 75 years 75 – 85 years > 85 years		*Ref* 12 (158) (0.942) (-298, 321) 9 (191) (0.963) (-366, 384) 116 (219) (0.598) (-314, 545) -225 (353) (0.523) (-916, 466)
Gender	Male Female		208* (112) (0.064) (-12, 427) *Ref*
Marital Status	Married or Cohabiting Other		-47 (216) (0.826) (-470, 375) *Ref*
Education Level	Primary Education Secondary Education: Intermediate Secondary Education: Leaving Certificate Third Level: Diploma/Certificate Third Level: Primary Degree Third Level: Postgraduate/Higher Degree		*Ref* 430** (155) (0.005) (127, 733) 533*** (160) (0.001) (219, 847) 738*** (185) (0.000) (375, 1101) 690*** (213) (0.001) (274, 1107) 952*** (272) (0.000) (420, 1484)
Employment	Retired Employed Other		*Ref* -299* (166) (0.071) (-624, 26) 90 (143) (0.526) (-189, 370)
Medical Card	No Medical Card Full Medical Card GP Visit Medical Card		*Ref* 478*** (145) (0.001) (193, 762) 47 (317) (0.883) (-575, 668)
Private Health Insurance	No Private Health Insurance Private Health Insurance		*Ref* 174 (123) (0.157) (-67, 415)
Region	Dublin City or County Other Town or City Rural area		*Ref* 156 (138) (0.261) (-116, 427) 188 (131) (0.150) (-68, 444)
Nationality	Irish Non-Irish		47 (171) (0.782) (-287, 382) *Ref*
Living Arrangement	Living Alone Living with Spouse Living with Others		*Ref* -3 (259) (0.992) (-511, 506) 66 (205) (0.747) (-335, 467)
Self-Rated Health	Excellent Very Good Good Fair Poor		*Ref* 136 (286) (0.636) (-426, 697) 597** (276) (0.031) (55, 1138) 1501*** (284) (0.000) (944, 2059) 2227*** (310) (0.000) (1620, 2834)
N GLM Family/Link AIC Modified Park Test Chi_Squared P-value Pearson Correlation Test Pregibon Link Test		4,342 Poisson/Log 3621.493 1.113506 1.0000 1.0000 1.0000	4,342 Poisson/Log 3282.652 1.369725 0.8727 0.7613 0.3287

***p<0.01, ** p<0.05, *p<0.10.

In terms of gender, males had additional healthcare costs of €160 (95% CIs: 16, 285) relative to females. Compared to a primary education, those with a secondary education (intermediate) were associated with additional costs of €235 (95% CIs: 49, 422), while those with a leaving certificate education were associated with additional costs of €291 (95% CIs: 84, 498). Costs were higher for those with a third level education. For instance, a third level primary degree education was associated with additional costs of €443 (95% CIs: 224, 664). In terms of health insurance status, a full medical card was associated with additional costs of €340 (95% CIs: 167, 513) and private health insurance was associated with additional costs of €184 (95% CIs: 32, 335). Finally, relative to excellent self-assessed health, good health was associated with additional costs of €753 (95% CIs: 465, 1042); fair health was associated with additional costs of €1455 (95% CIs: 1149, 1760); and poor health was associated with additional costs of €1971 (95% CIs: 1615, 2327).

In the multivariable analysis for the multimorbidity estimation sample (Table 4), chronic disease status, gender, education status, employment status, medical card status and self-reported health status were significantly associated with costs. Relative to those reporting two chronic conditions, four chronic conditions were associated with additional costs of €411 (95% CIs: 106, 716), five chronic conditions were associated with additional costs of €591 (95% CIs: 214, 969), and six or more chronic conditions were associated with additional costs of €1006 (95% CIs: 641, 1371). Compared to those reporting a primary education only, those reporting a secondary education (intermediate) education were associated with additional costs of €430 (95% CIs: 127, 733), while those reporting a third level primary degree education were associated with additional costs of €690 (95% CIs: 274, 1107). A full medical card was associated with additional costs of €478 (95% CIs: 193, 762). Finally, relative to excellent self-assessed health, good health was associated with additional costs of €597 (95% CIs: 55, 1138); fair health was associated with additional costs of €1501 (95% CIs: 944, 2059); and poor health was associated with additional costs of €2227 (95% CIs: 1620, 2834).

The results from the supplementary analyses are presented in Appendix 5. In the multivariable analyses for GP costs, hospital admission costs, outpatient clinic costs, and accident and emergency costs, the estimates revealed consistent and statistically significant associations between multimorbidity status and costs. Notably, the most economically meaningful impacts were observed for hospitalisation costs, followed by outpatient clinic costs and GP costs, which were similar in magnitude, and finally accident and emergency department costs. In the gender specific analyses, the cost estimates were marginal greater for males than for females. Notably, in the age category analyses, there was no statistically significant evidence of associations between multimorbidity status and total healthcare costs for the 75-85 age group or for the over 85 years group. Alternatively, there were statistically significant findings for each of the other age categories.

## Discussion

Multimorbidity is a growing public health issue globally, which presents significant challenges for health systems, payers, patients and their families.^1-4,10-12^ Moreover, the economic literature highlights that the burden of multimorbidity increases with the number of co-occurring chronic conditions experienced.^13-16^ In Ireland, a number of studies have explored the relationship between multimorbidity and costs of care using a variety of data sources and from a variety of perspectives,^17,18^ and have generally found positive and significant relationships. Our work builds upon this earlier research by further exploring the relationship between the number of additional chronic conditions and direct healthcare costs for sample cohorts with and without multimorbidity.

We find that, from a healthcare system perspective, relative to those with no chronic conditions, a single chronic condition was associated with additional annual costs of €513 (95% CIs: 245, 781) on average. Multiple chronic conditions were associated with additional average costs ranging from €610 (95% CIs: 346, 873) for two conditions to €1277 (95% CIs: 942, 1612) for six or more conditions. Further, within the multimorbidity cohort, compared to those reporting two chronic conditions, four chronic conditions were associated with additional annual costs of €411 (95% CIs: 106, 716), five conditions with additional costs of €591 (95% CIs: 214, 969), and six or more conditions with additional annual costs of €1006 (95% CIs: 641, 1371). Notably, the costs estimates were generally greater in magnitude for males than females, and were only significant for those in age categories up to the age of 75 years. While beyond the scope of our analysis, this latter finding may align with literature by authors such as Howdon and Rice,^37^ suggesting that it is proximity to death, rather than morbidity or multimorbidity status, which becomes the key determinant of healthcare costs in the 75 years and older age categories. Finally, the most economically meaningful impacts were observed for hospitalisation costs, followed by outpatient clinic costs and GP costs. These findings may be useful for informing the design and delivery of policy and practice in the context of multimorbidity care. In particular, these data may form part of the evidence base used to inform the evaluation of health technologies, as well as health and social care programmes and interventions, which target multimorbidity in Ireland and internationally. That is, our estimates enable an economic case to be made for interventions and programmes that go to prevent or delay the onset of multimorbidity and additional chronic conditions, and the potential range of cost savings that healthcare systems may expect to experience should such interventions or programmes prove to be effective. Further, our estimates go to support the argument that proactive preventive approaches, which require upfront financial investment, may ultimately prove to be cost effective, given their potential health benefits and cost savings to healthcare systems.

Our findings build on those of previous Irish and international literature. In the Irish context, our findings supplement those of Larkin, Walsh,^18^ which focused on the relationship between multimorbidity and patients’ out-of-pocket healthcare expenditures, and utilised TILDA wave 4 data. The study found that multimorbidity status, presented in the form of counts of two conditions and three or more conditions, led to significant out-of-pocket expenditures across a wide range of healthcare services, and highlighted that this financial burden may impact access to essential health care services for those with greatest need. After adjusting for covariates, relative to no conditions, having 3 or more conditions was associated with higher expenditures in absolute terms of €425. Having 2 conditions was associated with higher expenditures in absolute terms of €231. Our findings, while based on data from an earlier wave of TILDA, build upon this work by presenting estimates of the direct healthcare system costs related to multimorbidity, which are borne collectively by both public and private payers. Further, we were able to estimate the impact of additional chronic conditions on costs relative to reference categories of no chronic conditions and two chronic conditions. Our analysis is more similar in focus and form to that of Glynn, Valderas,^17^ which estimated the direct healthcare costs associated with multimorbidity, presented in the form of counts of 2, 3, and 4 or more conditions, based on a sample of 3,309 patients living in the community in the west of Ireland. After multivariate adjustment for age, gender and medical care eligibility, the addition of each chronic condition led to an associated annual increase in total healthcare costs (€4,096.86 versus €760.20 for >4 conditions versus 0 conditions). Our paper represents a complement and an update on this analysis, albeit using an alternative and nationally representative dataset, and more comprehensive statistical analysis with a larger set of independent variables. Looking to the international evidence base, our findings reflect those from multiple sources, which highlight that the healthcare burden attributable to multimorbidity increases as the number of co-occurring chronic conditions increases.^13-16^ In particular, our results for the average cost increases for those reporting four, five, or six or more chronic conditions within the multimorbidity sample cohort, complement those found in systematic reviews by Lehnert, Heider^15^ and Soley-Bori, Ashworth^16^ respectively. Notably, we do not find evidence of an exponential growth in costs with the burden additional chronic conditions. Overall, our findings go to further highlight the healthcare cost savings that potentially exist through proactive interventions that may reduce potentially unnecessary healthcare utilisation among those with multiple conditions.^16^ Nonetheless, robust evidence on the safety, clinical and cost effectiveness of such interventions within the multimorbidity patient population would be required before their implementation in clinical practice.

The study had a number of limitations. A key limitation is the cross-sectional nature of the study design, which stipulates that our findings must be interpreted as associations rather than causal effects. That said, TILDA includes a large and nationally representative sample of community-dwelling adults in Ireland. In future work, we plan to exploit additional waves of the dataset to explore transitions into multimorbidity categories and their resultant impacts on healthcare costs, but this was beyond the scope of this analysis. While missing data were not a significant concern, it may be the case that healthcare utilisation and costs may differ for those excluded from the analysis; thereby potentially biasing our results. A further limitation is that our total healthcare cost dependent variable and our key multimorbidity independent variable were generated on the basis of self-reported data, which are prone to recall bias. These issues generally give rise to concerns over the accuracy of our estimates. As stated above, a related limitation was that the available resource use data in the public database for each service was truncated. While we do not perceive this to be a major issue, it may indicate that our mean costs are underestimated. Given the limited numbers in highest resource categories, we did not employ statistical methods, such as those proposed by Parker and Fenwick^38^ to account for truncation. This may be viewed as a limitation. We also use TILDA wave 1 data, which was collected over 10 years ago, as it provides count data from all the resources of interest, and it provides sufficient participant numbers to allow for a more comprehensive multimorbidity status count variable of up to 6 or more conditions, thereby adding to the existing literature which was limited to maximums of 3 or more (Larkin et al) and four or more (Glynn et al) conditions respectively. That said, and while we submit that our findings are unlikely to be significantly impacted, it may be argued that our cost estimates are representative of a pre-pandemic population as the pattern of chronic disease is likely to have changed in the aftermath of the pandemic.^39^ This may give rise in some quarters to concerns over the applicability of our estimates to inform current policy debates. Relatedly, our total cost variable is limited in that it only includes the combined costs of GP and hospital inpatient, outpatient, and A&E utilisation. While we argue that these constitute a significant component of healthcare service delivery, it is important to acknowledge that the absence of other healthcare costs, such as those relating to the use of medications, present a significant omission from our analysis. Indeed, given the relationship between multimorbidity and polypharmacy, our findings are likely to underestimate the healthcare system impact of multimorbidity ^40^. As stated above, the exclusion of these services was pragmatically informed by the nature of the data available for analysis. Notably, the work by Larkin, Walsh,^18^ based on wave four of TILDA was able to capture out-of-pocket expenditures on many of these services. Our findings may be viewed as supplemental to those from this earlier study. Further, we employed available unit cost estimates for the healthcare services included, which may be viewed as mean estimates and therefore not reflective the variation that may exist with respect to the costs of care. In terms of our key independent variable, there are legitimate arguments over the use of disease counts to capture multimorbidity. In particular, this approach does not directly consider the relative severity of each of the conditions that the individual experiences. While we do attempt to control for this somewhat in our regression analysis, it remains unclear the extent to which our approach addresses this issue. The inclusion of self-reported health as an independent variable may be problematic if it captures part of the multimorbidity impact, thereby leading our findings to underestimate that multimorbidity impact. We present results in Appendix 5 from regression models which exclude self-reported health as an explanatory variable, and which report higher estimates for the costs relating to multimorbidity. Nonetheless, as per the Andersen model, the ‘need’ factors influencing healthcare utilisation and costs include both actual and perceived health status.^26^ The inclusion of multimorbidity status, informed by the diagnosis of a doctor, and self-rated health status in our base case models seeks to address both the former and the latter respectively. Relatedly, while we include a wide range of independent variables in our regression analysis, a case could be made for those missing variables excluded, such as objective measures of health, severity of illness and income (which was subject to significant missing data in TILDA wave 1), which may go to undermine the presented coefficient estimates. In respect of our multivariable regression analysis, it should be noted that the analysis of cost data is by its nature problematic. For example, our total healthcare cost dependent variable exhibited high levels of skewness (i.e. 4.83) and kurtosis (i.e. 33.67). In this regard, we employ GLM regression approaches in an attempt to explicitly account for the distributional nature of our cost dependent variable. In respect of the above listed limitations, we have endeavoured at all times to be careful and pragmatic in the choices we have made and cautious in how we have interpreted our findings, both in the context of Ireland and their generalisability to international settings.

## Conclusion

In conclusion, this study adds to existing evidence base by exploring the relationship between multimorbidity and direct healthcare system costs for older people in Ireland. Our findings may be of relevance to patients and their families, charities, healthcare providers, policy makers and researchers, who are interested in articulating the economic case for the investment in health technologies and health and social interventions which target the prevention and management of multimorbidity in Ireland and internationally.

## Supplemental Material

Supplemental Material - Exploring the link between Multimorbidity and direct healthcare costs in Ireland: A cross-sectional study Click here for additional data file. Supplemental Material for Exploring the link between Multimorbidity and direct healthcare costs in Ireland: A cross-sectional study by Sharon Walsh and Paddy Gillespie in Journal of Multimorbidity and Comorbidity

## Note

1. Survey questions relating to resource use are provided in Appendix 2.
