# Supplementary material for: Exploring the link between Multimorbidity and direct healthcare costs in Ireland: A cross-sectional study
Source: J Multimorb Comorb. 2023 Dec 10;13:26335565231219421. doi: 10.1177/26335565231219421 (PMC10712276; doi:10.1177/26335565231219421)
Supplement: Supplemental Material - Exploring the link between Multimorbidity and direct healthcare costs in Ireland: A cross-sectional study [file sj-pdf-1-cob-10.1177_26335565231219421.pdf]

**APPENDIX 1: SURVEY QUESTIONS**

***Health Condition Questions***

PH201: Please look at card PH1. Has a doctor ever told you that you have any of the conditions on this card?

1. High blood pressure or hypertension GO TO PH202 [ph201_1]
2. Angina GO TO PH203 [ph201_2]
3. A heart attack (including myocardial infarction or coronary thrombosis) GO TO PH205 [ph201_3]
4. Congestive heart failure GO TO PH212 [ph201_4]
5. Diabetes or high blood sugar GO TO PH213 [ph201_5]
6. A stroke (cerebral vascular disease) GO TO PH218 [ph201_6]
7. Ministroke or TIA GO TO PH221 [ph201_7]
8. High cholesterol GO TO PH225 [ph201_8]
9. A heart murmur GO TO PH301 [ph201_9]
10. An abnormal heart rhythm GO TO PH301 [ph201_10]
95. Any other heart trouble (specify) [ph201a] GO TO PH224 [ph201_11]
96. None of these GO TO PH301 [ph201_14]
98. DK GO TO PH301 [ph201_12]
99. RF GO TO PH301 [ph201_13]

(ELSA/ similar questions in HRS/ SHARE)

PH301: Please look at card PH2. Has a doctor ever told you that you have any of the following conditions?

1. Chronic lung disease such as chronic bronchitis or emphysema GO TO PH302 [ph301_1]
2. Asthma [ph301_2]
3. Arthritis (including osteoarthritis, or rheumatism) GO TO PH304 [ph301_3]
4. Osteoporosis, sometimes called thin or brittle bones [ph301_4]
5. Cancer or a malignant tumour GO TO PH309 [ph301_5] (including leukaemia or lymphoma but excluding minor skin cancers)
6. Parkinson's disease GO TO PH314 [ph301_6]
7. Any emotional, nervous or psychiatric problems, such as depression or anxiety GO TO PH315 [ph301_7]
8. Alcohol or substance abuse GO TO PH320 [ph301_8]
9. Alzheimer's disease GO TO PH318 [ph301_9]
10. Dementia, organic brain syndrome, senility GO TO PH319 [ph301_10]
11. Serious memory impairment GO TO PH319a [ph301_11]
12. Stomach ulcers [ph301_12]
13. Varicose Ulcers (an ulcer due to varicose veins) [ph301_13]
14. Cirrhosis, or serious liver damage [ph301_14]
96. None of these [ph301_17]
98. DK [ph301_16]
99. RF [ph301_15]

(ELSA/ similar question HRS/NSHAP)

***Resource Use Questions***

**HU005:** In the last 12 months, about how often did you visit your GP?
*[IWER: IF RESPONDENT HAS NOT VISITED GP IN THE LAST 12 MONTHS CODE 0]*

**HU008:** In the last 12 months, about how many visits did you make to a hospital as an out-patient? (Include all types of consultations, tests, operations, procedures or treatments)
*[IWER: IF RESPONDENT HAS NOT MADE ANY OUT-PATIENT VISITS, CODE 0]*

**HU010:** In the last 12 months, on how many occasions were you admitted to hospital overnight?
*[IWER: IF RESPONDENT HAS NOT ADMITTED TO HOSPITAL OVERNIGHT IN THE LAST 12 MONTHS CODE 0]*

**HU012:** In total, about how many nights did you spend in hospital in the last 12 months?

**HU007:** In the last 12 months, how often did you visit a hospital Emergency Department (sometimes called A&E or Accident and Emergency) as a patient?
*[IWER: IF RESPONDENT HAS NOT VISITED AN A&E DEPARTMENT IN THE LAST 12 MONTHS CODE 0]*

***<u>Other Relevant Survey Questions</u>***

**AGE:** Age at interview assuming DOB is 1st of specified month
Age at interview assuming DOB is 1st of specified month

**SEX:** Gender
Gender

**CS006:** Are you...?
[FOR THE FIRST INTERVIEW READ OUT] Can I just check again, are you...[OTHERWISE] Are you...
1 Married
2 Living with a partner as if married
3 Single (never married)
4 Separated
5 Divorced
6 Widowed

**DM001:** What is the highest level of education you have completed?

Now I would like to ask some questions about your background. What is the highest level of education you have completed?
1 Some primary (not complete)
2 Primary or equivalent

3 Intermediate/junior/group certificate or equivalent
4 Leaving certificate or equivalent
5 Diploma/certificate
6 Primary degree
7 Postgraduate/higher degree
96 None
98 Don't know

**WE001:** Which one of these would you say best describes your current situation?
*[IWER: SHOW CARD WE1]*

Now I'm going to ask you some questions about work, retirement and pensions. Please look at card WE1. Which one of these would you say best describes your current situation? *[IWER: CODE THE ONE THAT APPLIES]*
1 Retired
2 Employed
3 Self-employed (including farming)
4 Unemployed
5 Permanently sick or disabled
6 Looking after home or family
7 In education or training
95 Other (Specify)
98 Don't know

**DM011:** Were you born in the Republic of Ireland?

Were you born in the Republic of Ireland?
1 Yes
5 No

**HU001:** Are you covered by...
1. Full Medical Card or equivalent
2. GP Visit Card
96. Neither of these
98. Don’t Know
99. Refused

*[IWER: CODE THE ONE THAT APPLIES] [NOTE: THIS QUESTION IS ASKED EVEN OF THOSE COVERED BY PRIVATE MEDICAL INSURANCE. MOST OVER 70S ARE ENTITLED TO MEDICAL CARDS.]*

**HU002:** Do you have private medical insurance cover (VHI etc.) in your own name or through another family member?
1. Yes, in my own name
2. Yes, as the spouse of a subscriber
3. Yes, as the relative of a subscriber
5 No
98. Don’t Know
99. Refused

**LOCAL3:** Location of household - Dublin/Urban/Rural
Location of household - Dublin/Urban/Rural
1 Dublin city or county
2 Another town or city
3 A rural area

**SOCLIVESWITH5:** Individual level household composition
Individual level household composition
1 Living alone
2 Living with spouse only
3 Living with child/step/adopted/grandchild
4 Living with other relative
5 Living with unrelated ppl

**PH001:** Now I would like to ask you some questions about your health. Would you say?

[IWER: CODE THE ONE THAT APPLIES] Now I would like to ask you some questions about your health. Would you say your health is..
1 Excellent
2 Very good
3 Good
4 Fair
5 Poor

STROBE Statement—checklist of items that should be included in reports of observational studies

| | Item No. | Recommendation | Page No. | Relevant section in manuscript |
|---|---|---|---|---|
| **Title and abstract** | 1 | (*a*) Indicate the study's design with a commonly used term in the title or the abstract | 2 | Yes, this is presented in the Abstract and in the Methods sections |
| | | (*b*) Provide in the abstract an informative and balanced summary of what was done and what was found | 2 | Yes |
| **Introduction** | | | | |
| Background/rationale | 2 | Explain the scientific background and rationale for the investigation being reported | 3 and 4 | Yes, this is presented in the Introduction section |
| Objectives | 3 | State specific objectives, including any pre-specified hypotheses | 4 | Yes, this is presented in the Background section |
| **Methods** | | | | |
| Study design | 4 | Present key elements of study design early in the paper | 5 | Yes, this is presented in the Methods section |
| Setting | 5 | Describe the setting, locations, and relevant dates, including periods of recruitment, exposure, follow-up, and data collection | 5 | Yes, this is presented in the Methods section |
| Participants | 6 | (*a*) *Cohort study*—Give the eligibility criteria, and the sources and methods of selection of participants. Describe methods of follow-up<br>*Case-control study*—Give the eligibility criteria, and the sources and methods of case ascertainment and control selection. Give the rationale for the choice of cases and controls<br>*Cross-sectional study*—Give the eligibility criteria, and the sources and methods of selection of participants | 5 | Yes, this is presented in the Methods section |
| | | (*b*) *Cohort study*—For matched studies, give matching criteria and number of exposed and unexposed<br>*Case-control study*—For matched studies, give matching criteria and the number of controls per case | N/A | N/A |
| Variables | 7 | Clearly define all outcomes, exposures, predictors, potential confounders, and effect modifiers. Give diagnostic criteria, if applicable | 6,7 and 8 | Yes, this is presented in the Methods section |
| Data sources/ measurement | 8* | For each variable of interest, give sources of data and details of methods of assessment (measurement). Describe comparability of assessment methods if there is more than one group | 6,7 and 8 | Yes, this is presented in the Methods section |
| Bias | 9 | Describe any efforts to address potential sources of bias | | Yes, this is presented in the Methods section and the resulting limitations in the Discussion section |

| | | | | |
|---|---|---|---|---|
| Study size | 10 | Explain how the study size was arrived at | 8, 13-14 | Yes, this is presented in the Methods section |

Continued on next page

| | | | | |
|---|---|---|---|---|
| Quantitative variables | 11 | Explain how quantitative variables were handled in the analyses. If applicable, describe which groupings were chosen and why | 8,9 | Yes, this is presented in the Methods section |
| Statistical methods | 12 | (*a*) Describe all statistical methods, including those used to control for confounding | 8,9 | Yes, this is presented in the Methods section |
| | | (*b*) Describe any methods used to examine subgroups and interactions | 9 | Yes, this is presented in the Methods section |
| | | (*c*) Explain how missing data were addressed | 5,6 | Yes, this is presented in the Methods section. Missing data was trivial in this case |
| | | (*d*) *Cohort study*—If applicable, explain how loss to follow-up was addressed<br>*Case-control study*—If applicable, explain how matching of cases and controls was addressed<br>*Cross-sectional study*—If applicable, describe analytical methods taking account of sampling strategy | 8 | This is presented in the Methods section. Data were analysed as presented in TILDA. |
| | | (*e*) Describe any sensitivity analyses | 9 | This is presented in the Methods section, with the focus on supplementary analysis. |
| **Results** | | | | |
| Participants | 13* | (a) Report numbers of individuals at each stage of study—eg numbers potentially eligible, examined for eligibility, confirmed eligible, included in the study, completing follow-up, and analysed | 9, 20,21 | The numbers included in the study are detailed in Table 1 and in the Results section |
| | | (b) Give reasons for non-participation at each stage | | N/A |
| | | (c) Consider use of a flow diagram | | N/A |
| Descriptive data | 14* | (a) Give characteristics of study participants (eg demographic, clinical, social) and information on exposures and potential confounders | 9, 20,21 | Yes, this is presented in the Results section and in Table 1 and in the Appendix |
| | | (b) Indicate number of participants with missing data for each variable of interest | | N/A |
| | | (c) *Cohort study*—Summarise follow-up time (eg, average and total amount) | | N/A |
| Outcome data | 15* | *Cohort study*—Report numbers of outcome events or summary measures over time | | *N/A* |
| | | *Case-control study*—Report numbers in each exposure category, or summary measures of exposure | | *N/A* |
| | | *Cross-sectional study*—Report numbers of outcome events or summary measures | 9, 20,21, 22 | Yes, this is presented in the Results section, Table 1 and Table 2 |
| Main results | 16 | (*a*) Give unadjusted estimates and, if applicable, confounder-adjusted estimates and their precision (eg, 95% confidence interval). Make clear which confounders were adjusted for and why they were included | 23, 24 | Yes, this is presented in the Results section, Table 3 and Table 4 |

| | | | |
|---|---|---|---|
| | (*b*) Report category boundaries when continuous variables were categorized | 23,24 | Yes, this is presented in the Results section, Table 3 and Table 4 |
| | (*c*) If relevant, consider translating estimates of relative risk into absolute risk for a meaningful time period | | N/A |

Continued on next page

| | | | | |
|---|---|---|---|---|
| Other analyses | 17 | Report other analyses done—eg analyses of subgroups and interactions, and sensitivity analyses | 35-38 | Yes, this is presented in the Appendix Section |
| **Discussion** | | | | |
| Key results | 18 | Summarise key results with reference to study objectives | 11, 12 | Yes, this is presented in the Discussion section |
| Limitations | 19 | Discuss limitations of the study, taking into account sources of potential bias or imprecision. Discuss both direction and magnitude of any potential bias | 13, 14 | Yes, this is presented in the Discussion section |
| Interpretation | 20 | Give a cautious overall interpretation of results considering objectives, limitations, multiplicity of analyses, results from similar studies, and other relevant evidence | 14. 15 | Yes, this is presented in the Discussion section |
| Generalisability | 21 | Discuss the generalisability (external validity) of the study results | 14 | Yes, this is presented in the Discussion section |
| **Other information** | | | | |
| Funding | 22 | Give the source of funding and the role of the funders for the present study and, if applicable, for the original study on which the present article is based | 1 | Yes, this is presented in the Title page |

**APPENDIX 3:** Descriptive Statistics on Individual Chronic Conditions

| **Chronic Conditions** | **Full Estimation Sample**<br><br>**N (%)**<br>**8,447** | **Multimorbidity Estimation Sample**<br>**N (%)**<br>**4,342** |
|---|---|---|
| High Blood Pressure | 3,058 (36.20) | 2,535 (58.38) |
| Angina | 447 (5.29) | 435 (10.02) |
| Heart Attack | 377 (4.46) | 350 (8.06) |
| Congestive Heart Failure | 89 (1.05) | 86 (1.98) |
| Diabetes | 632 (7.48) | 569 (13.10) |
| Stroke | 131 (1.55) | 116 (2.67) |
| Transient Ischaemic Attack | 174 (2.06) | 167 (3.85) |
| High Cholesterol | 3,160 (37.41) | 2,569 (59.17) |
| Heart Murmur | 408 (4.83) | 357 (8.22) |
| Abnormal Heart Rhythm | 592 (7.01) | 538 (12.39) |
| Other Heart Trouble | 295 (3.49) | 270 (6.22) |
| Chronic Lung Disease | 333 (3.94) | 300 (6.91) |
| Asthma | 769 (9.10) | 657 (15.13) |
| Arthritis | 2,269 (26.86) | 1,930 (44.45) |
| Osteoporosis | 788 (9.33) | 686 (15.80) |
| Cancer | 521 (6.17) | 443 (10.20) |
| Parkinson’s Disease | 43 (0.51) | 35 (0.81) |
| Emotion, Nervous, Psychiatric Problems including Anxiety or Depression | 721 (8.54) | 591 (13.61) |
| Alcohol or Substance Abuse | 132 (1.56) | 119 (2.74 |
| Serious Memory Impairment | 38 (0.45) | 33 (0.76) |
| Stomach Ulcer | 581 (6.88) | 500 (11.52) |
| Varicose Ulcers | 276 (3.27) | 238 (5.48) |
| Cirrhosis or serious liver damage | 46 (0.54) | 42 (0.97) |

**APPENDIX 4:** Unit cost estimates

| Resource Activity | Activity | Unit Cost 2020 (€) prices | Source of Estimates |
|---|---|---|---|
| General Practitioner (GP) Clinic | per visit | 51 | Smith et al (2021), Survey of Private GP Fees, 2019 € prices |
| Hospital Inpatient Admission | per admission | 4555 | Hospital Pricing Office, Direct Request, 2015 € prices |
| Outpatient Clinic Consultation | per visit | 136 | Hospital Pricing Office, Direct Request, 2015 € prices |
| Accident and Emergency Department | per visit | 264 | Hospital Pricing Office, Direct Request, 2015 € prices |

† Unit costs in 2020 prices. Unit costs were inflated using the health component of the consumer price index [24]

**APPENDIX 5:** Supplementary Analyses

**Appendix 5 - Table 1:** Marginal effect coefficients from generalized linear regression models of **primary care, hospital admissions, outpatient clinic consultations, and accident and emergency healthcare costs (€)**, estimated using a Poisson error distribution and a log link function, based on the full estimation sample, and estimated controlling for full set of independent variables listed above

| Variable Name | Variable Description<br>Chronic Condition Status | Multivariable Model<br>€ Coeff (SE) (P-Value) (95% CIs) |
|---|---|---|
| Primary Care | No chronic conditions | *Ref* |
| | 1 chronic condition | 69 (8) (0.000) (55, 84) |
| | 2 chronic conditions | 103 (8) (0.000) (88, 118) |
| | 3 chronic conditions | 113 (8) (0.000) (97, 129) |
| | 4 chronic conditions | 124 (9) (0.000) (106, 142) |
| | 5 chronic conditions | 141 (11) (0.000) (120, 162) |
| | 6 or more chronic conditions | 160 (12) (0.000) (137, 183) |

| | | |
|---|---|---|
| Hospital Admission | No chronic conditions<br>1 chronic condition<br>2 chronic conditions<br>3 chronic conditions<br>4 chronic conditions<br>5 chronic conditions<br>6 or more chronic conditions | *Ref*<br>360 (132) (0.006) (101, 619)<br>389 (131) (0.003) (133, 645)<br>421 (143) (0.003) (142, 701)<br>554 (147) (0.000) (265, 842)<br>646 (168) (0.000) (318, 975)<br>863 (160) (0.000) (550, 1177) |
| Outpatient Clinic Consultations | No chronic conditions<br>1 chronic condition<br>2 chronic conditions<br>3 chronic conditions<br>4 chronic conditions<br>5 chronic conditions<br>6 or more chronic conditions | *Ref*<br>66 (13) (0.000) (41,91)<br>104 (13) (0.000) (79,129)<br>129 (14) (0.000) (102, 156)<br>147 (15) (0.000) (119, 176)<br>172 (16) (0.000) (140, 203)<br>190 (16) (0.000) (159, 222) |
| Accident and Emergency Department Visits | No chronic conditions<br>1 chronic condition<br>2 chronic conditions<br>3 chronic conditions<br>4 chronic conditions<br>5 chronic conditions<br>6 or more chronic conditions | *Ref*<br>19 (7) (0.010) (4, 33)<br>15 (7) (0.048) (0.2, 29)<br>26 (8) (0.002) (9, 42)<br>36 (9) (0.000) (18, 54)<br>40 (10) (0.000) (20, 59)<br>58 (10) (0.000) (38, 77) |

*** p<0.01, ** p<0.05, *p<0.10.

**Appendix 5 - Table 2:** Marginal effect coefficients from generalized linear regression models of **total healthcare costs (€) by gender**, estimated using a Poisson error distribution and a log link function, based on the full estimation sample, and estimated controlling for full set of independent variables listed above

| **Variable Name** | **Variable Description**<br>**Chronic Condition Status** | **Multivariable Model**<br>**€ Coeff (SE) (P-Value) (95% CIs)** |
|---|---|---|
| Male<br>N= 3,756 | No chronic conditions<br>1 chronic condition<br>2 chronic conditions<br>3 chronic conditions<br>4 chronic conditions<br>5 chronic conditions<br>6 or more chronic conditions | *Ref*<br>761 (200) (0.000) (368, 1153)<br>711 (199) (0.000) (321, 1101)<br>923 (224) (0.000) (484, 1362)<br>1078 (232) (0.000) (624, 1533)<br>1305 (271) (0.000) (774, 1837)<br>1392 (251) (0.000) (900, 1883) |
| Female<br>N= 4,691 | No chronic conditions<br>1 chronic condition<br>2 chronic conditions<br>3 chronic conditions<br>4 chronic conditions<br>5 chronic conditions<br>6 or more chronic conditions | *Ref*<br>316 (189) (0.094) (-54, 685)<br>529 (184) (0.004) (169, 890)<br>547 (196) (0.005) (164, 930)<br>737 (210) (0.000) (327, 1148)<br>766 (238) (0.001) (300, 1232)<br>1209 (237) (0.000) (744, 1674) |

*** p<0.01, ** p<0.05, *p<0.10.

**Appendix 5 – Table 3:** Marginal effect coefficients from generalized linear regression models of **total healthcare costs (€) by age category**, estimated using a Poisson error distribution and a log link function, based on the full estimation sample, and estimated controlling for full set of independent variables listed above

| **Variable Name** | **Variable Description**<br>**Chronic Condition Status** | **Multivariable Model**<br>**€ Coeff (SE) (P-Value) (95% CIs)** |
|---|---|---|
| <55 years | No chronic conditions | *Ref* |
| N= 1,946 | 1 chronic condition | 395 (187) (0.035) (29, 761) |
| | 2 chronic conditions | 710 (182) (0.000) (354, 1066) |
| | 3 chronic conditions | 765 (225) (0.001) (323,1207) |
| | 4 chronic conditions | 892 (274) (0.001) (355, 1429) |
| | 5 chronic conditions | 702 (310) (0.024) (95,1309) |
| | 6 or more chronic conditions | 1400 (266) (0.000) (880, 1921) |
| 55 – 65 years | No chronic conditions | *Ref* |
| N= 3,022 | 1 chronic condition | 372 (196) (0.058) (-12, 755) |
| | 2 chronic conditions | 538 (195) (0.006) (156, 920) |
| | 3 chronic conditions | 377 (221) (0.088) (-57, 810) |
| | 4 chronic conditions | 897 (227) (0.000) (452, 1341) |
| | 5 chronic conditions | 866 (263) (0.001) (350, 1382) |
| | 6 or more chronic conditions | 977 (247) (0.000) (493, 1462) |
| 65 – 75 years | No chronic conditions | *Ref* |

| | | |
|---|---|---|
| N= 2,146 | 1 chronic condition<br>2 chronic conditions<br>3 chronic conditions<br>4 chronic conditions<br>5 chronic conditions<br>6 or more chronic conditions | 974 (301) (0.001) (383, 1564)<br>733 (282) (0.009) (181, 1286)<br>782 (302) (0.010) (190, 1373)<br>910 (312) (0.004) (298, 1521)<br>1304 (351) (0.000) (616, 1991)<br>1613 (355) (0.000) (917, 2309) |
| 75 – 85 years<br>N= 1,104 | No chronic conditions<br>1 chronic condition<br>2 chronic conditions<br>3 chronic conditions<br>4 chronic conditions<br>5 chronic conditions<br>6 or more chronic conditions | *Ref*<br>293 (650) (0.653) (-982, 1567)<br>415 (637) (0.515) (-834, 1664)<br>1163 (663) (0.080) (-137, 2463)<br>955 (680) (0.160) (-378, 2288)<br>1036 (708) (0.143) (-352, 2423)<br>1393 (681) (0.041) (59, 2727) |
| > 85 years<br>N= 229 | No chronic conditions<br>1 chronic condition<br>2 chronic conditions<br>3 chronic conditions<br>4 chronic conditions<br>5 chronic conditions<br>6 or more chronic conditions | *Ref*<br>-707 (882) (0.423) (-2435, 1021)<br>-847 (1011) (0.402) (-2829,1135)<br>-761 (1060) (0.473) (-2838, 1316)<br>-436 (1181) (0.712) (-2751, 1880)<br>469 (949) (0.621) (-1391, 2329)<br>747 (931) (0.422) (-1078, 2573) |

*** p<0.01, ** p<0.05, *p<0.10.

**Appendix 5 – Table 4:** Marginal effect coefficients from generalized linear regression models of healthcare costs (€), estimated using a Poisson error distribution and a log link function, based on the full estimation sample, and excluding self-rated health as an independent variable

| **Variable Name** | **Variable Description** | **Multivariable Model**<br>**€ Coeff (SE) (P-Value) (95% CIs)** |
|---|---|---|
| Chronic Condition Status | No chronic conditions<br>1 chronic condition<br>2 chronic conditions<br>3 chronic conditions<br>4 chronic conditions<br>5 chronic conditions<br>6 or more chronic conditions | *Ref*<br>693 (138) (0.000) (423, 963)<br>991 (133) (0.000) (729, 1252)<br>1186 (140) (0.000) (911, 1461)<br>1512 (152) (0.000) (1213, 1810)<br>1807 (170) (0.000) (1473, 2141)<br>2189 (167) (0.000) (1862, 2516) |
| N<br>GLM Family/Link<br>AIC<br>Modified Park Test<br>Pearson Correlation Test<br>Pregibon Link Test<br>Modified Hosmer and Lemeshow | | 8,447<br>Poisson/Log<br>3058.162<br>0.990437<br>0.8666<br>0.8466<br>0.3401 |

*** p<0.01, ** p<0.05, *p<0.10.

**Appendix 5 – Table 5:** Marginal effect coefficients from generalized linear regression models of healthcare costs (€), estimated using a Poisson error distribution and a log link function, based on the multimorbidity estimation sample, and excluding self-rated health as an independent variable

| **Variable Name** | **Variable Description** | **Multivariable Model**<br>**€ Coeff (SE) (P-Value) (95% CIs)** |
|---|---|---|
| Chronic Condition Status | 2 chronic conditions | *Ref* |
| | 3 chronic conditions | 281 (140) (0.045) (7, 556) |
| | 4 chronic conditions | 737 (158) (0.000) (428, 1046) |
| | 5 chronic conditions | 1117 (187) (0.000) (750, 1484) |
| | 6 or more chronic conditions | 1668 (183) (0.000) (1309, 2026) |
| N | | 4,342 |
| GLM Family/Link | | Poisson/Log |
| AIC | | 3519.257 |
| Modified Park Test | | 1.414179 |
| Pearson Correlation Test | | 0.9402 |
| Pregibon Link Test | | 0.9073 |
| Modified Hosmer and Lemeshow | | 0.9942 |

*** p<0.01, ** p<0.05, *p<0.10.
